# Elevated Serum Interleukin-8 Level as a Preferable Biomarker for Identifying Uncontrolled Asthma and Glucocorticosteroid Responsiveness

**Published:** 2017-06

**Authors:** Jingxi Zhang, Chong Bai

**Affiliations:** Department of Respiratory and Critical Care Medicine, Changhai Hospital, the Second Military Medical University, Shanghai, China

**Keywords:** Uncontrolled asthma, Interleukin-8, Fractional exhaled nitrite oxide, Biomarker

## Abstract

**Background:**

To explore the clinical significance of serum interleukin-8 (IL-8) level as a biomarker for uncontrolled asthma in order to improve our understanding of asthma phenotypes and facilitate the development of new therapeutic agents in the future.

**Materials and Methods:**

A total of 246 uncontrolled asthma patients and 50 healthy controls were selected from an outpatient clinic during October 2015 and April 2016. The clinical data were collected, and the levels of IL-8, IL-6, tumor necrosis factor-α (TNF-α), and immunoglobulin (IgE) were measured in peripheral blood via ELISA assay. The level of serum IL-8 was compared between the glucocorticosteroid groups, receiving inhaled corticosteroids (ICs), oral corticosteroids (OCs), and intravenous corticosteroids (GCs), respectively. Changes in the serum IL-8 level were compared between asthmatics with good and poor glucocorticosteroid responsiveness.

**Results:**

The serum IL-8 level in uncontrolled asthmatics (87.45 pg/mL; 5–7500) was significantly higher than that of the healthy controls (10.9 pg/mL; 6.8–39.65; *P*< 0.001). The increase in the serum IL-8 level above the normal range occurred in 58.13% of uncontrolled asthmatics. The area under curve (AUC) for serum IL-8 level, indicative of uncontrolled asthma, was 0.816 (95% CI, 0.7605 to 0.8721; *P*< 0.0001), which was greater than the AUC of fractional exhaled nitric oxide (AUC, 0.711; 95% CI, 0.6057 to 0.8153; *P*= .0188). The serum IL-8 level showed a significant positive relationship with blood neutrophil count (*P*= 0.0004), neutrophil percentage (*P*= 0.027), serum TNF-α protein (*P*< 0.0001), forced expiratory volume/forced vital capacity (FEV_1_/FVC) ratio (*P*< 0.05), and rate of FEV_1_ change after bronchodilation. The level of IL-8 in patients requiring OCs or GCs treatment was significantly higher than that of ICs patients (186 and 235 pg/mL vs. 61 pg/mL; *P*< 0.0001). The reduction in the serum IL-8 level was more significant in asthmatic patients with good responsiveness (277 pg/mL (65.3–3124) to 67.8 pg/mL (5–1408); *P*< 0.0001), compared to those with poor responsiveness (218 pg/mL (64.8–7500) vs. 197 pg/mL (56.9–5238); *P*= 0.49).

**Conclusion:**

The increase in serum IL-8 level can be used as a preferable biomarker to identify asthma status and initial treatment in asthmatics. The change in IL-8 level also reflects the response to glucocorticosteroids in uncontrolled asthma. These exploratory results suggest an association between the pathophysiology, inflammation, and clinical outcomes of asthma. This raises the possibility of developing new agents for IL-8 inhibition and helps provide more precise and personalized asthma care.

## INTRODUCTION

Asthma is a common respiratory disease, characterized by chronic airway inflammation dominated by T helper 2 (Th2) cells. Millions of patients still suffer from this incurable disease, and the control rate of asthma is unsatisfactory worldwide according to epidemiological investigations (1, 2). Improvement of the overall asthma control and timely and effective alleviation of symptoms are becoming major clinical problems, which need to be addressed urgently. Proper control of the disease depends on both detection of uncontrolled cases and acceptable response to available asthma medicines. Therefore, search for rapid and convenient biomarkers for evaluation of control conditions and prediction of treatment responsiveness should be resolved in clinics abruptly.

Over the past decades, there have been great interests in the role of inflammation in the pathogenesis and pathophysiology of asthma. Accumulating evidence suggests that cytokine alterations and proinflammatory state are associated with asthma (3–5). However, research on the role of systematic proinflammatory mediators is more limited than studies on airway local inflammation, and the available data originate from case studies with a small number of samples or controversial results.

Interleukin-8 (IL-8) is considered an important chemotactic factor, involving neutrophil recruitment and activation; it is also active on primed eosinophils. It can be secreted by many structural and immune cells, including bronchial epithelial cells, smooth muscle cells, and macrophages. Some studies have reported increased serum levels of IL-8 during asthma attacks and allergic dermatitis (6). IL-8 level also increases in the induced sputum or bronchoalveolar lavage fluid of asthmatics, suggesting the involvement of IL-8 in the pathogenesis of inflammatory and allergic diseases (7, 8).

Brown et al. (9) recently reported that serum Th17-associated cytokines, including IL-8, were positively associated with difficult-to-control asthma in inner-city African American children. The association of severe asthma with increased IL-8 level in the sputum or bronchoalveolar lavage fluid has been previously reported in adults and children (10, 11). The literature suggests that IL-8 plays an important role in refractory neutrophilic asthma and is an indicator of neutrophilic phenotypes in asthma patients.

As a potent proinflammatory cytokine, the function and role of serum IL-8 in adult asthma have not been yet fully examined. The present study was designed to explore the potential of serum IL-8 in determining the disease status and to describe its significance as an indicator of treatment responsiveness in a series of uncontrolled adult asthma patients. This study was conducted in order to deepen our understanding of asthma phenotypes and to provide a framework for the development of new target therapeutic agents in the future.

## MATERIALS AND METHODS

### Subjects

This observational, case-control study was conducted between October, 2015 and April, 2016 in the outpatient clinic of the Department of Respiratory and Critical Care Medicine, Changhai Hospital. A total of 246 uncontrolled asthma patients above 18 years were diagnosed by a physician according to the Global Initiative for Asthma (GINA) criteria. The uncontrolled asthma condition was evaluated according to the GINA standards (12). Patients with an acute attack or at least 3 of the following manifestations in the past 4 weeks were defined as uncontrolled asthma: 1) daytime symptoms more than twice a week; 2) nocturnal awakening due to asthma; 3) relievers needed more than twice a week; and 4) any activity limitations due to asthma (12).

The clinical data of each patient, including the symptoms, atopic comorbidities, family history, and Asthma control Test (ACT) scores, were collected at the time of recruitment. None of the subjects had a history of smoking. The characteristics of the patients are presented in Table 1. Fifty healthy subjects were selected as the controls. Subjects were excluded if they had any other known pulmonary diseases, including chronic obstructive pulmonary disease (COPD) or any major comorbidities with possible effects on asthma activity (eg, HIV, metastatic cancer, diabetes, and congestive heart failure). This study was approved by the Ethics Committee of Changhai Hospital, and a signed informed consent form was obtained from each participant.

**Table 1.** The clinical characteristics of the subjects included in the study

**Parameters**	**Uncontrolled asthma**	**Healthy control**
Age (years)	51.9(14.3)	51.5(14.5)
Male number (female)	102(144)	28(22)
Asthma duration (Years)	2.5(0.15–40)	None
ACT scores	15.6(2.57)	None
Allergic rhinitis	55.28%(136/246)	None
Blood neutrophil counts (×10^9^/L)	4.05(1.81–13.7)	3.67(2.75–5.48)
Blood neutrophil percentage (%)	61.00(40.1–90.9	63.2(53.9–72)
Blood eosinophil counts (×10^9^/L)	0.15(0.01–6.95)	0.1(0.04–0.17)^**^
Blood eosinophil percentage (%)	2.4(0.2–47.2)	1.5(0.6–3.2)^*^
IgE (IU/ml)(<165)	41.2(5–2662)	
Pre-FEV_1_/FVC	76.75(15.33)	84.92(5.58)^*^
Post-FEV_1_/FVC	77.00(15.30)	85.85(5.60) ^*^
Pre-FEV_1_(L)	2.39(0.97)	2.8(0.75) ^*^
Post-FEV_1_(L)	2.52(0.96)	2.98(0.72) ^*^
Pre-FEV_1_% predictated	91.53(29.29)	110.47(18.64) ^**^
Post-FEV_1_%% predictated	97.52(28.69)	117.76(16.55) ^**^
Pre-FEF25–75% predictated	72.53(39.35)	98.19(22.48) ^**^
Post-FEF25–75% predictated	78.09(39.07)	109.76(24.63) ^**^
Pre-FEF50% predictated	75.33(42.32)	103.69(25.53) ^**^
Post-FEF50% predictated	83.00(42.46)	117.76(27.22) ^**^
FENO(ppb)	22(5–300)	17(8–26) ^*^
SOD(U/ml)	157.73(22.98)	206.75(21.26) ^*^

**Note:**

* *P*<0.05 vs control group,

** *P*<0.01 vs control group,

**Abbreviations:** ACT scores, asthma control test scores; FEV_1_, forced expiratory volume in one second; FVC, forced vital capacity; FEF, forced expiratory flow; FENO, fractional exhaled nitric oxide.

### Pulmonary function test (PFT) and fractional exhaled nitric oxide (FeNO) measurements

The bronchial challenge test (BCT) or bronchodilator test is used to diagnose asthma. PFT was performed using a pneumotachograph-based system in accordance with the recommendations of the European Respiratory Society (13). The FeNO level was determined, using the NIOX MINO chemiluminescence analyzer (Aerocrine AB, Solna, Sweden), according to the guidelines established by the American Thoracic Society (ATS) (13). The patients were asked to inhale the maximum amount of air and were then instructed to exhale air into the valve connected to the analyzer. The flow rate (50 mL/s) was kept constant, and data were recorded after 90 seconds; all these procedures were followed-up by the clinician.

### Serum component measurements

The blood stored in EDTA tube supernatants (5 cc of blood vein sample) was assayed to determine the levels of IL-8, tumor necrosis factor-α (TNF-α), and IL-6 via ELISA assay (Diaclone, France), following the manufacturer’s instructions. At lower detection limits of 5, 4, and 2 pg/mL, the upper normal limitations were 62, 8.1, and 5.9 pg/mL, respectively. All the blood supernatants were stored at −70°C before assays. Total immunoglobulin (IgE; normal range < 165 IU/mL) was assayed by ELISA (EU-Immune, Germany). The complete blood cell count (CBC) with differentiation was also assayed. The level of superoxide dismutase (SOD) was assayed using the ELISA kit, with a normal range of 129–216 U/mL.

### Glucocorticosteroid treatment responsiveness

The patients were categorized in 3 steps, based on the short-term step-up GINA strategy (12) until relieving the symptoms. The first step included inhaled corticosteroid (ICs)/long-acting β2-agonist (LABA; salmeterol/fluticasone, 50/250 or 50/500; GSK Company, UK) twice a day for 2 weeks (ICS group). After repeating the ACT, if the patient still complained of the symptoms and had an ACT score below 20, the second step was initiated with 30 mg of prednisone (XINYI Company, Shanghai, China) once a day for 5–7 days in combination with ICS/LABA (OCS group).

If the patient complained of the symptoms and had an ACT score below 20, the third step was initiated with 40 mg of intravenous methylprednisolone (Pifer Company, USA) per day for 3–5 days (GCS group). After a month of treatment, an ACT score > 20 in patients with a high IL-8 level was defined as good glucocorticosteroid responsiveness; otherwise, it was defined as poor responsiveness.

### Statistical analysis

Graphpad Prism 5 was used for data analysis. Continuous variables are presented as mean (standard deviation) and median (minimum and maximum), while categorical variables are presented as a percentage (case number/total number). Continuous variables were tested by Mann–Whitney U test for nonparametric data and independent *t* test for parametric data. Dichotomous variables were tested by Fisher’s exact test or Chi square test. The serum IL-8 levels in the figures were transformed to a natural logarithm to mitigate the influence of extreme outliers. For determining the correlation between different variables, Spearman’s correlation test was used. Comparison of IL-8 and FeNO measurements was also conducted by plotting ROC curves and measuring the area under curve (AUC). *P*-value < 0.05 indicated statistical significance.

## RESULTS

### Clinical characteristics of uncontrolled asthma

A total of 246 uncontrolled asthma patients were enrolled in this study. The clinical characteristics and demographic profile of the subjects are presented in Table 1. The major symptoms of uncontrolled asthma included chest tightness (97.56%; 240/246), shortness of breath (95.93%; 236/246), fatigue usually after exertion (74.39%; 183/246), heart palpitation (65.85%; 162/246) especially at night, and persistent or transient typical wheezing (57.31%; 141/246). Other atypical symptoms included belching (49.18%; 121/246), insomnia (43.08%; 106/246), chest pain (31.7%; 78/246), and cough (26.82%; 66/246). Compared to the healthy controls, higher serum levels of eosinophil (*P*< 0.05) and FeNO (*P*< 0.001) were reported in patients with uncontrolled asthma, and lung function decreased significantly, as indicated by the predicted reduction in forced expiratory volume/forced vital capacity (FEV_1_/FVC) ratio, FEV_1_, forced expiratory flow (FEF 25–75%), and FEF 50%.

### Increased serum level of IL-8 in uncontrolled asthma patients compared to the control group

**Figure 1.**
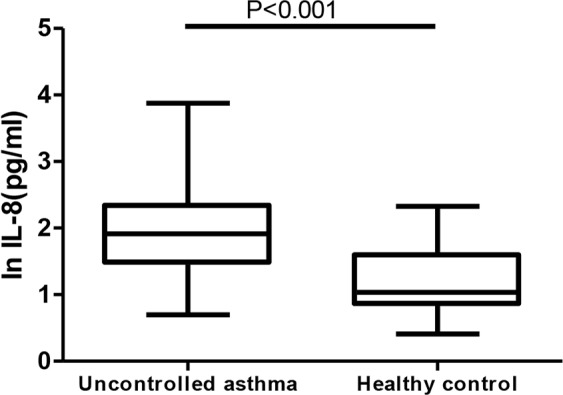
Comparison of serum IL-8 level between uncontrolled asthma and healthy control. Box and whisker plots represent medians, interquartile ranges, and range.

As illustrated in Figure 1, the serum IL-8 level in uncontrolled asthma patients (87.45 pg/mL; 5–7500) was significantly higher than that of the healthy controls (10.9 pg/mL; 6.8–39.65) (*P*< 0.001). The median of serum IL-8 level was 87.45 pg/mL (5–7500), which exceeded the normal range (< 62 pg/mL) and indicated an increase in serum IL-8 level in uncontrolled asthma. The serum levels of TNF-α were 6.22 pg/mL (2.6–18.4) and 7.1 pg/mL (6.4–7.6) in patients and controls, respectively, while IL-6 levels were 2.78 pg/mL (2–44.71) and 2.8 pg/mL (2.1–5.3). However, no significant difference was found in these mediators between the groups.

### Elevated serum IL-8 level indicative of the high incidence of uncontrolled asthma

**Figure 2.**
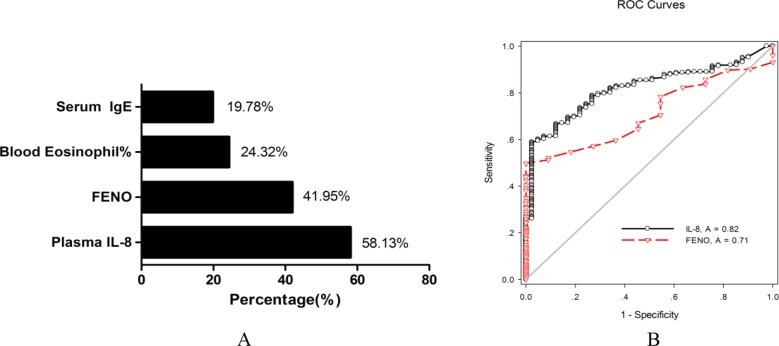
Comparison of incidence (A) and efficacy (B) of elevated serum IL-8 and other asthmatic biomarker in uncontrolled asthma patients. FENO, fractional exhaled nitric oxide.

In all uncontrolled asthma patients, the incidence of elevation in serum IL-8 level (> 62 pg/mL) was 58.13% (143/246). In all 246 patients, the incidence of elevation in common asthma biomarkers was 41.95% for FeNO, 24.32% for blood eosinophil percentage, and 19.78% for serum IgE level (Figure 2A). The incidence of increase in TNF-α and IL-6 levels in all 246 patients was 23.58% (58/246) and 13.00% (32/246), respectively. The number of patients with a high IL-8 level (> 62 pg/mL; 143/246, 58.13%) was higher than that of patients with a high FeNO level (> 25ppb; 86/205; 41.95%; χ^2^, 10.78; *P*< 0.01). The AUC of serum IL-8 level indicating uncontrolled asthma was 0.816 (95% CI, 0.7605 to 0.8721; *P*< 0.0001), which was greater than that of FeNO (AUC, 0.711; 95% CI, 0.6057 to 0.8153; *P*= .0188); however, the difference was insignificant (*P*= 0.05121; Figure 2B).

### Relationship between serum IL-8 level and blood neutrophils and airflow reversibility in uncontrolled asthma patients

There was a significant relationship between serum IL-8 level and blood neutrophil count (R^2^, 0.089; *P*= 0.0004), neutrophil percentage (R^2^, 0.035; *P*= 0.027), serum TNF-α protein (R^2^, 0.1958; *P*< 0.0001), FEV_1_/FVC ratio (R^2^, 0.05076; *P*= 0.0103), and rate of FEV_1_ change after bronchodilation (R^2^, 0.05668; *P*= 0.0073; Table 2). However, no significant relationship was found between serum IL-8 and IL-6 levels and allergic indicators, including serum IgE, FeNO, blood eosinophils, blood eosinophil percentage, and other pulmonary function parameters.

**Table 2.** The relationship between of serum IL-8 and other clinical parameters of 246 uncontrolled asthma patients

**Parameters**	**r^2^**	**P value**
Blood neutrophil counts(×10^9^/L)	0.08939	**0.0004**
Blood neutrophil percentage (%)	0.03532	**0.0273**
TNF-α(pg/ml)(<8.1)	0.1958	**< 0.0001**
IL-6(pg/ml)(<5.9)	0.01359	0.1083
Post-FEV_1_/FVC	0.05076	**0.0103**
The change rate of FEV_1_ after bronchodilation	0.05668	**0.0073**

### High serum IL-8 level related to the high dosage of glucocorticosteroids in uncontrolled asthma

**Figure 3.**
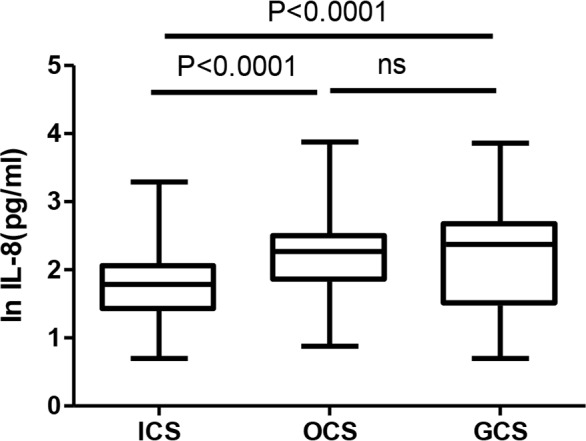
The elevated serum IL-8 in different glucocorticosteroid treatment groups.

All the patients were initially prescribed ICs/LABA inhalation. Nonetheless, 15 (15/246; 6.10%) patients left the study and could not be followed-up for their clinical data. The percentage of effective treatment with ICs, oral corticosteroids (OCs), and intravenous corticosteroids (GCs) was 45.61%, 26.31%, and 28.07%, respectively in the high IL-8 group over the past month versus 70% (χ^2^, 8.3; *P*< 0.01), 10% (χ^2^, 5.51; *P*< 0.05), and 20% in the low IL-8 group. The ICs, OCs, and GCs groups comprised 53.24% (123/231), 20.77% (48/231), and 25.97% (60/231) of the study sample, respectively. Age, asthma duration, IL-6, FeNO, and serum superoxide dismutase (SOD) levels were higher in the GCs group, compared to the ICs group (*P*= 0.0173, *P*= 0.0009, *P*= 0.045, *P*= 0.0237, and *P*= 0.0054, respectively; Table 3). Compared with the ICs group (61 pg/mL, 5–1957), the median of serum IL-8 level in the OCs (186 pg/mL, 7.52–7500) and GCs (235 pg/mL, 5–7227) groups was significantly higher (*P*< 0.0001; Figure 3).

**Table 3.** The differences of clinical features between ICS, OCS and GCS group

	ICS(n=123)	OCS(n=48)	GCS(n=60)	P value^*^
Age (years)	48(16–83)	58.5(26–76)	59.5(20–76)^*^	**0.0173**
Asthma duration (years)	1.5(0.1–40)	2.5(0.1–20)	5.0(0.1–50)^*^	**0.0009**
Plasma IL-8 (pg/ml)	61(5–1957)	186(7.52–7500)	235(5–7227)	**0.0001**
Plasma TNF-a (pg/ml)	6.005(2.6–11.1)	6.6(4–16.2)	6.28(4–18.4)	ns
Plasma IL-6 (pg/ml)	2.52(2–9.85)	2.92(2–12.8)	3.2(2–44.71)^*^	**0.045**
FENO (ppb)	17(5–174)	23(5–162)	30(5–145)^*^	**0.0237**
Serum SOD (U/ml)	165(130–239)	154(122–243)	147(87–179)^*^	**0.0054**

*: Compared with ICS group

### Significant reduction of serum IL-8 level in uncontrolled asthma patients with good glucocorticosteroid responsiveness

**Figure 4.**
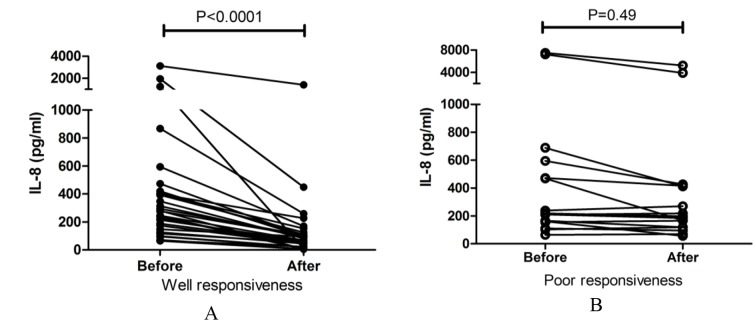
The change of elevated serum IL-8 between well (A) and poor (B) glucocorticosteroid responsiveness in asthma patients.

The ACT score was measured in a total of 110 (110/143; 76.92%) patients with a high serum IL-8 level after 1 month of glucocorticosteroid treatment. Overall, 78 patients showed good glucocorticosteroid responsiveness, as indicated by the increase in ACT score from 15.9 (1.95) to 23.46 (0.96) (*P*< 0.001), while 33 cases showed poor responsiveness, as indicated by the increase in ACT score from 15.05 (2.69) to 19.47 (1.86). The serum IL-8 level decreased by 77.5% after treatment, compared to the pretreatment period in patients with good responsiveness (277 pg/mL (65.3–3124) vs. 67.8 pg/mL (5–1408); *P*< 0.0001) (Figure 4A), while it reduced by 9.6% in the group with poor responsiveness, without any significant difference (218 pg/mL (64.8–7500) vs. 197 pg/mL (56.9–5238), *P*= 0.49) (Figure 4B).

## DISCUSSION

In this study, a higher incidence of increase in serum IL-8 level was observed in comparison with other common biomarkers in uncontrolled asthma, regardless of the attack or chronic persistent state of asthma. The results showed that patients with a high level of IL-8 required higher doses of glucocorticosteroids for the initial treatment, and a rapid decline in the elevated serum IL-8 level indicated good response to drugs. To the best of our knowledge, this is the first survey of the clinical significance of serum IL-8 level from diagnostic and prognostic perspectives in many asthmatics.

In general, IL-8 is a well-known type of classic chemokine, involved in neutrophil recruitment and activation (14). The increase in IL-8 level has been shown to play a pivotal role in different allergic or autoimmune disorders, such as allergic dermatitis (15), Henoch-Schonlein purpura (16), allergic rhinitis (17), allergic conjunctivitis (18), and autoimmune hepatitis (19, 20). Asthma is also a disorder, associated with dysimmunoregulation and Th1/Th2 cell imbalance due to atopy.

The increase in serum IL-8 level in our study substantiated evidence on the importance of IL-8 in allergy-related diseases. Previous studies have paid particular attention to IL-8 expression in bronchoalveolar lavage, showing that IL-8 level increases specifically in severe asthma (21) and is inversely correlated with FEV_1_ (22). Wouters et al. (23) introduced systemic inflammation as a key factor in the pathogenesis of asthma and COPD. The origin of systemic inflammation was generally considered the spillover of inflammatory mediators into circulation (inflammatory spillover phenomenon). Our results indicated an obvious IL-8-related systemic inflammation in some asthma patients, suggesting not only local airway inflammation, but also innate systemic immunity activation in asthma.

Search for a sensitive and convenient biomarker to identify uncontrolled asthmatics is an essential measure to improve asthma control. FeNO level was frequently measured to distinguish between airway eosinophilic and non-eosinophilic inflammation (24). The overall specificity was higher than its sensitivity, indicating a higher diagnostic potential for diagnosing rather than ruling out asthma (25). In this study, patients with a normal FeNO and elevated IL-8 were not uncommon. Generally, mere reliance on FeNO measurements may lead to the missed diagnosis of asthma and inadequate assessment, which is in line with the mentioned findings (25). Moreover, it is difficult and unrealistic to detect inflammatory mediators by inducing sputum or repeating bronchoscopy to evaluate asthma status.

As mentioned earlier, IL-8 level elevates in various diseases in suspicious or diagnosed patients with typical or atypical asthma symptoms. Since nearly 60% of patients in our series showed an increase in IL-8 level (greatly higher than the percentage of elevated IgE, eosinophils, TNF-α, or IL-6) and AUC of IL-8 increase was even higher than that of FeNO, IL-8 elevation could be considered a valuable biomarker for accurate identification of uncontrolled asthma with confirmed sensitivity and specialty. It is also a convenient indicator, which can be easily applied and developed in clinics. Lack of a relationship between IL-8 and allergic markers, such as blood eosinophil count and IgE, shows that IL-8 may be a potential marker for nonallergic asthma, also known as endogenous asthma.

In clinics, if correct diagnosis or treatment is delayed or irregular, asthma may gradually progress to a severe state, even to fixed airflow obstruction due to decreased airway reversibility. The related mechanism may be involved in airway remodeling and proliferation of airway smooth muscles. Previous studies found that IL-8 level contributes to the pathogenesis of severe asthma by directly facilitating airway remodeling, increasing the migration and proliferation of bronchial smooth muscle cells (26), and inducing airway hyperresponsiveness (27), angiogenesis (28), epithelial-mesenchymal transition in the airway (29), and neutrophil recruitment.

In this study, we first reported that serum IL-8 level was inversely correlated with pulmonary function and airway reversibility reduction, thereby suggesting the important role of IL-8 in the progress of fixed obstructive ventilation dysfunction in uncontrolled asthma. Early intervention targeting IL-8 may be helpful in inhibiting the deterioration of lung function. On the other hand, Ghaffari et al. (30) reported no difference in IL-8 level between mild and severe asthmatics, while Silvestri et al. (31) reported that serum IL-8 level was higher in severe asthma patients than mild asthma controls; the discrepancy in the results may be related to the number of patients in each study.

IL-8 elevation was associated with the activation of nuclear factor (NF)-κB molecular pathway (32). Glucocorticosteroid, as the most effective NF-κB inhibitor, could reportedly reduce IL-8 production in the literature. Our previous experiments showed that IL-8 expression of human epithelial cells induced by poly(I:C), a classic toll-like receptor 3 (TLR3) ligand, could be inhibited by dexamethasone in a dose-dependent manner; therefore, the IL-8 increase resulted from NF-κB pathway activation.

The increase in IL-8 level contributes to neutrophil infiltration and activation by producing neutrophil elastases. In a previous study, neutrophilic asthma was reported as a mechanism of glucocorticosteroid resistance or insensitivity. Liu et al. (33) found that NF-κB-mediated IL-8 expression may be involved in steroid-resistant airway inflammation, stimulated by the multi-allergen challenge of asthma in an animal model and experiments on airway epithelial cells *in vitro*. Also, a higher dose of drugs is needed in neutrophilic asthma. Our results showed that the initial dose of glucocorticosteroids was higher in patients with a high level of IL-8, compared to those with a normal level of IL-8. Therefore, IL-8 may help physicians prescribe adequate amounts of antiasthma drugs so as to improve treatment compliance and promptly gain better control.

Based on our results, the condition of patients with a slow reduction in IL-8 level or a rapid decline in IL-8 did not improve. It was concluded that some asthmatics with high IL-8 levels showed insensitivity to regular asthma drugs, and velocity of IL-8 reduction could predict the treatment outcomes for uncontrolled asthma. A new phenotype can be assigned to these patients as the high IL-8 subgroup, characterized by the high dose of drug and possibility of poor responsiveness to initial treatment.

IL-8 can be a valuable biomarker for defining the specific pathophysiology of different asthma phenotypes and identifying potential therapeutic targets. The mechanism may be related to serum SOD reduction and TNF-α involvement, considering the possible relationship between systematic oxidative stress (34) or other proinflammatory meditors (35) and glucocorticosteroid insensitivity.

CXCR2 is one of the receptors for IL-8 (36). A recent study demonstrated that CXCR2 inhibitor reduced the sputum neutrophilia and showed a trend towards exacerbation and improvement of Asthma Control Questionnaire (ACQ) score in patients with severe asthma (37). Agents targeting CXCR2 or IL-8 signal pathways should be explored as alternative therapeutic strategies in uncontrolled asthmatics with elevated neutrophils so as to avoid the side effects of high-dose glucocorticosteroids.

Application of all auxiliary tests should be first based on the patient’s complaints. In this study, wheezing, as a typical symptom of asthma, was only reported in half of the patients. During the study, we found different misdiagnoses in patients without wheezing symptoms. We also found that long-term heart palpitation might be a major complaint of asthma patients, which is worthy of attention in clinics.

The limitations of the present study include the short observational time and incomplete data acquisition. Moreover, no analysis of sputum was carried out to distinguish the cellular phenotype in patients. Therefore, it is not convincing to confirm the relationship between serum IL-8 level and precise phenotypes in asthma. This study mostly focused on the clinical importance of IL-8 in patients with initially diagnosed or poorly controlled asthma and evaluated the prediction of treatment responsiveness. The significance of serum IL-8 level can be more convincing if the data of controlled asthmatic patients are compared accurately in further investigations.

In conclusion, we found that the level and trend of increase in serum IL-8 level could be used as a preferable biomarker for uncontrolled asthma and glucocorticosteroid responsiveness in asthmatics (especially in a long period). These findings can be helpful in distinguishing certain patient populations, who are more likely to respond to ICs. This study also presents a theoretical framework for the development of new agents for IL-8 inhibition in order to provide more personalized and specialized asthma care for this subgroup of patients in the future.
